# Identification of Vibration Frequencies of Railway Bridges from Train-Mounted Sensors Using Wavelet Transformation

**DOI:** 10.3390/s23031191

**Published:** 2023-01-20

**Authors:** Emrah Erduran, Fredrik Marøy Pettersen, Semih Gonen, Albert Lau

**Affiliations:** 1 Department of Built Environment, Oslo Metropolitan University, 0176 Oslo, Norway; 2 Department of Civil and Environmental Engineering, Norwegian University of Science and Technology, 7034 Trondheim, Norway

**Keywords:** vehicle-track-bridge interaction, bridge frequency, drive-by monitoring, vehicle scanning method, wavelet transformation, dynamic identification

## Abstract

This article presents a novel methodology to extract the bridge frequencies from the vibrations recorded on train-mounted sensors. Continuous wavelet transform is used to distinguish the bridge frequencies from the other peaks that are visible in the Fourier amplitude spectrum of the accelerations recorded on train bogies. The efficacy of the proposed method is demonstrated through numerical case studies. For this, a detailed three-dimensional finite element model that can capture the vibration characteristics of the bridge, track, and train is created, and each component of the model is separately validated. The train model used is a three-dimensional multi-degree-of-freedom system that can simulate the pitching and rolling behavior. The train was then virtually driven over the bridge at different speeds and under varying track irregularities to evaluate the robustness of the proposed method in extracting bridge frequencies from train-mounted sensors under different conditions. The proposed methodology is shown to be capable of identifying bridge modal frequencies even for aggressive track irregularity profiles and relatively high speeds of trains.

## 1. Introduction

Identifying the dynamic properties of bridges including their vibration frequencies is critical for understanding their behavior. This will allow engineers to, among other things, create numerical models that can better represent the physical behavior of the bridge [1,2,3], identify any degradation in the materials by tracking the variations in the frequencies over time [4], and determine the critical speeds of the trains that traverse the railway bridges with the ultimate goal of effective and efficient infrastructure management [5,6,7,8].

Conventionally, identification of frequencies and mode shapes is based on on-site measurements, which requires instrumentation of each bridge using several accelerometers and a data acquisition system (e.g., [9,10]). These methods are referred to as direct measurement methods as they directly measure the vibrations on the bridge [11]. One of the most significant limitations of direct measurement methods is the need for a separate instrumentation system for each bridge. Considering the sheer number of bridges that are in operation worldwide, the costs and the labor-intensive nature of instrumenting each bridge with its own instrumentation system becomes quickly overwhelming. Thus, only a fraction of the bridges is currently instrumented [12].

Using vehicle-mounted sensors to identify the vibration frequencies of the bridges provides an attractive alternative. These methods promise to identify the vibration frequencies of numerous bridges from a few sensors mounted on a vehicle at a fraction of the cost compared to the direct methods. The idea of using indirect methods, known as such because the vibrations on the bridge are never measured directly, was first proposed in [13] and several studies have been published on this topic since. The focus of the early studies was the identification of highway bridge frequencies using the accelerations obtained from the quarter- or half-car models in numerical simulations [14,15,16,17,18,19]. Several other studies then aimed to determine the mode shapes of bridges [20,21,22,23,24,25,26], to identify and remove the adverse effects of surface roughness [27,28,29,30,31], or to identify the presence of damage [32,33,34,35,36]. Although few in number, laboratory and field experiments were used to confirm the theoretical and numerical findings [37,38,39,40,41,42,43]. Refs. [11,12] provide an excellent summary of the most influential publications on the indirect methods. At the end of these summaries, Yang et al. [11,12] also list the challenges that still remain on the identification of dynamic properties of bridges via indirect measurements. The three most prominent challenges are as follows: (i) Most studies are based on vehicle models that are simplified as a single-degree-of-freedom (SDOF) system. Few studies so far have considered multiple DOFs that represent the vehicle and the coupling effect of the multi-DOFs such as pitching and rolling. (ii) The Fourier Amplitude Spectrum (FAS) of the accelerations recorded on a vehicle-mounted sensor was shown, both analytically and experimentally [13,37], to include the vibration frequencies of the bridge. However, these spectra often include numerous other peaks due to road roughness and other factors, whose effects can arguably be alleviated using different methods such as using dual vehicles, filtering techniques, inclusion of vehicle damping, etc. (iii) Vehicle speed directly affects the duration of the data sampling and the quality of the indirect measurements. It is, therefore, suggested in most of the previous studies to use low-to-moderate speeds for the vehicles.

These challenges, which are valid for the road bridges, can arguably be amplified for the railway bridges because of the complex nature of the railway track and its interaction with a railway bridge. Although indirect methods have been available since the turn of the century, only in 2021 their feasibility for the railway bridges was proven analytically [44]. After deriving the theoretical equations which show that the frequencies of the rail-track system and the bridge are inherent in the acceleration response of the vehicle, Yang and Yang [44] demonstrate this using finite element analysis considering the impact of different parameters such as track irregularities and infinite boundary conditions of the track. However, the FAS includes several other peaks many of which can be much more prominent than the peaks associated with the bridge frequencies. In a theoretical study such as [44], the frequencies of the bridge are known *a priori* and can be identified among several peaks in the FAS. Yet, in practice where the bridge frequencies are unknown and sought after, it is often not possible to identify them among many peaks. Furthermore, the train is represented by a SDOF, which cannot consider several phenomena related to the movement of a rail vehicle such as pitching as well as the loading frequencies associated with different degrees of freedom crossing the sleepers. In this regard, the examples of indirect monitoring for railway bridges are very scarce and mostly based on numerical simulations. Zhan et al. [45,46] conducted numerical studies using a series of SDOF and two-axle MDOF vehicles to identify the dynamic response of simply supported railway bridges. Quirke et al. [47] tried to identify the presence of damage on railway bridges using a virtual longitudinal profile. Fitzgerald et al. [48] numerically investigated the feasibility of scour damage monitoring using the acceleration measurements obtained from train bogies. Matsuoka et al. [49] proposed a method to identify resonant railway bridges using the track irregularity measured from high-speed trains. They also applied their method to operating high-speed trains in Japan and demonstrated the resonance condition on some of the inspected railway bridges. It should be noted, however, that railway track was not explicitly considered in these studies.

The main contribution of this article is to propose a novel methodology that can distinguish bridge frequencies from the other frequencies visible on the Fourier amplitude spectrum. For this purpose, the proposed method combines Fourier Transform with Continuous Wavelet Transform (CWT). Leveraging the vibrations recorded on the bogie as it is crossing the bridge as well as the front and back approaches, the natural frequencies of the bridge are distinguished from other frequencies by investigating the time variation of the frequency content of the recorded vibrations using CWT. Hence, the article provides a theoretically sound and replicable methodology to identify the railway bridge frequencies using indirect methods considering track irregularities and relatively high train speeds. The proposed methodology is demonstrated on numerical case studies where the train is modeled using a three-dimensional multi-degree-of-freedom system with eight wheels located on four bogies. The bridge was modeled using a dual-beam system as suggested in [44] to fully capture the interaction between the vehicle, track and the bridge. Furthermore, the front and back approaches were modeled using a beam that simulates the rail with the ballast and the track stiffness is simulated using linear springs discretized at every 60 cm. We used two different track profiles to account for the effect of track irregularities on the frequency content of the vibrations recorded on the bogies. As such, the article addresses all three challenges in bridge frequency identification from indirect methods summarized in [11,12] by proposing a methodology that can distinguish bridge frequencies from others that are visible on a complicated Fourier amplitude spectra under both low and relatively high train speeds and using a complex vehicle model that can consider pitching and rolling effect.

The article is structured as follows: First, the theoretical formulation by [44] which proves that the bridge frequencies are visible in the acceleration response recorded on a vehicle traversing a railway bridge is summarized. A brief summary of the continuous wavelet transform is also introduced. In the following section, the breakdown of the proposed methodology is explained. In Section 4, a summary of the finite element model consisting of all the components of a railway system, i.e., the bridge, track, and the train is provided. Each component of the FE model is modeled in detail in three-dimensional space. The results of the numerical analysis carried out at different train speeds and with and without track irregularities are provided in the following section, where case studies are presented. The proposed methodology to detect the bridge frequencies from the vibrations recorded on the bogie of the train is demonstrated and its efficacy under different track irregularity profiles and train speeds is shown. Concluding remarks and suggestions for future works conclude the article.

## 2. Theoretical Background

### 2.1. Equation of Motion

**Figure 1:**
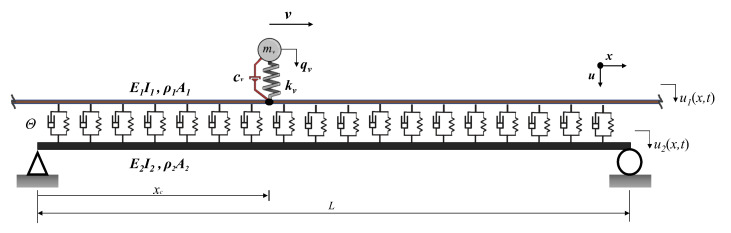
Sprung mass moving on a dual beam that represents the rail-track-bridge system.

The governing equations for a sprung mass traveling over a dual-beam system that simulates the rail-track-bridge structure (Figure 1) is presented in detail in [44] and will be summarized here. Assuming that the damping and the mass of the vehicle is very small compared to the track and the bridge, the governing equations of the rail and the bridge, i.e., the upper and lower beams, and the vehicle can be written as follows:

(1a) $$E_{1}I_{1}\frac{\partial^{4}u_{1}\left(x,t\right)}{\partial x^{4}}+\rho_{1}A_{1}\frac{\partial^{2}u_{1}\left(x,t\right)}{\partial x^{2}}+\Theta \left(u_{1}\left(x,t\right)-u_{2}\left(x,t\right)\right)=f_{c}\left(t\right)\delta \left(x-vt\right)$$

(1b) $$E_{2}I_{2}\frac{\partial^{4}u_{2}\left(x,t\right)}{\partial x^{4}}+\rho_{2}A_{2}\frac{\partial^{2}u_{2}\left(x,t\right)}{\partial x^{2}}+\Theta \left(u_{2}\left(x,t\right)+u_{1}\left(x,t\right)\right)=0$$

where subscripts 1 and 2 denote the beams representing the rail and the beam, respectively, ρ is the density, *E* is the Young´s modulus, *A* is the cross-sectional area, *I* moment of inertia and u(x,t) is the beam displacement, Θ is the track modulus, δ is the delta function.

(2a) $${\ddot{q}}_{v}\left(t\right)+w_{v}^{2}q_{v}\left(t\right)=w_{v}^{2}u_{1}\left(x_{c}\right)$$

(2b) $$f_{c}\left(t\right)=k_{v}\left(q_{v}-u_{1}\left(x_{c}\right)\right)+M_{v}g$$

where $q_{v}$ is the displacement of the vehicle, $w_{v}$ is its natural frequency, $M_{v}$ is the lumped vehicle mass, $k_{v}$ is the spring constant, $x_{c}$ is the coordinate of the contact point, $f_{c}$ is the contact force and *g* is the acceleration of gravity.

Defining $K_{i}=E_{i}I_{i}$, $M_{i}=\rho_{i}A_{i}$ ($i_{1},2$) and equating the right hand sides of Equation (1a), a couple of homogeneous differential equations that represent the free vibration behavior of the rail-bridge system is obtained:

(3a) $$K_{1}\frac{\partial^{4}u_{1}}{\partial x^{4}}+M_{1}\frac{\partial^{2}u_{1}}{\partial x^{2}}+\Theta \left(u_{1}-u_{2}\right)=0$$

(3b) $$K_{2}\frac{\partial^{4}u_{2}}{\partial x^{4}}+M_{2}\frac{\partial^{2}u_{2}}{\partial x^{2}}+\Theta \left(u_{2}+u_{1}\right)=0$$

Assuming that the mode shapes of a simply supported beam can be represented using sinusoidal functions and using modal superposition, the displacements $u_{i}\;\left(i=1,2\right)$ can be related to the modal coordinates $q_{in}$ as:

(4) $$u_{i}\left(x,t\right)=\sum_{n=1}^{\infty }\;q_{in}\left(t\right)sin\left(k_{n}x\right)$$

where $k_{n}=n\pi /L$.

Inserting Equation (4) into Equation (3) and solving these equations simultaneously, the natural frequencies of the beams representing the track and the bridge can be solved as:

(5a) $$w_{1n}^{2}=\frac{1}{2}\{\left(\Omega_{11n}^{2}+\Omega_{22n}^{2}\right)-\sqrt{{\left(\Omega_{11n}^{2}-\Omega_{22n}^{2}\right)}^{2}+4\Omega_{120}^{4}}\}$$

(5b) $$w_{2n}^{2}=\frac{1}{2}\{\left(\Omega_{11n}^{2}+\Omega_{22n}^{2}\right)+\sqrt{{\left(\Omega_{11n}^{2}-\Omega_{22n}^{2}\right)}^{2}+4\Omega_{120}^{4}}\}$$

where $\Omega_{iin}^{2}=\left(K_{i}k_{n}^{4}+\Theta \right)M_{i}^{-1}$, and $\Omega_{120}^{4}=\Theta^{2}{\left(M_{1}M_{2}\right)}^{-1}$.

With the frequencies of the dual-beam system available and using the modal superposition, the displacements of the upper and lower beams can be written as:

(6a) $$u_{1}\left(x,t\right)=\sum_{n=1}^{\infty }\;sin\left(k_{n}x\right)\sum_{i=1}^{2}S_{in}\left(t\right)$$

(6b) $$u_{2}\left(x,t\right)=\sum_{n=1}^{\infty }\;sin\left(k_{n}x\right)\sum_{i=1}^{2}a_{in}S_{in}\left(t\right)$$

where $S_{in}\left(t\right){=}_{in}sin\left(\omega_{in}t\right)+\eta_{in}cos\left(\omega_{in}t\right)$, ${}_{in},\;\eta_{in}$ are equation coefficients, and $a_{in}=\Omega_{10}^{-2}\left(\Omega_{11n}^{2}-\omega_{in}^{2}\right)$. Inserting Equation (6) into Equation (1), the equation of motion for the double beam system under a moving load can be rewritten as:

(7a) $$\sum_{n=1}^{\infty }\;sin\left(k_{n}x\right)\sum_{i=1}^{2}\left(\ddot{S_{in}}+\omega_{in}^{2}s_{in}\right)=M_{1}^{-1}f_{c}\left(x\right)\delta \left(x-vt\right)$$

(7b) $$\sum_{n=1}^{\infty }\;sin\left(k_{n}x\right)\sum_{i=1}^{2}\left(\ddot{S_{in}}+\omega_{in}^{2}s_{in}\right)a_{in}=0$$

Using the orthogonality rules and trigonometric functions, the closed-form solutions for the displacement of the dual-beam system subjected to a single moving load can be written as:

(8a) $$u_{1}\left(x,t\right)=\sum_{n=1}^{N}\sum_{i=1}^{2}\left(\frac{b_{ji}M_{v}g(sin\left(\omega_{ji}tvk_{i}-sin\left(k_{i}vt\right)\omega_{ji}\right)}{\omega_{ji}\left(vk_{i}-\omega_{}ji\right)\left(vk_{i}+\omega_{ji}\right)}\right)sin\left(k_{i}x\right)$$

(8b) $$u_{2}\left(x,t\right)=\sum_{n=1}^{N}\sum_{i=1}^{2}a_{ji}\left(\frac{b_{ji}M_{v}g(sin\left(\omega_{ji}tvk_{i}-sin\left(k_{i}vt\right)\omega_{ji}\right)}{\omega_{ji}\left(vk_{i}-\omega_{}ji\right)\left(vk_{i}+\omega_{ji}\right)}\right)sin\left(k_{i}x\right).$$

where *N* is the number of eigenmodes included, $b_{1n}=\frac{2a_{2n}}{\left(a_{2n}-a_{1n}M_{1}L\right)}$, $b_{2n}=\frac{2a_{1n}}{\left(a_{1n}-a_{2n}M_{1}L\right)}$

Using Equation (8a) and by setting x=vt and $u_{c}\left(t\right)=u_{1}\left(vt,t\right)$, the contact point displacement $u_{c}\left(t\right)$ can be computed as:

(9) $${\ddot{u}}_{c}\left(t\right)=\sum_{n=1}^{N}\sum_{i=1}^{2}A_{ij}cos\left(\left(\omega_{ji}-k_{i}v\right)t\right)+B_{ij}cos\left(\left(\omega_{ji}k_{i}v\right)t\right)+C_{ij}cos\left(2k_{i}vt\right)$$

$A_{ij},\;B_{ij},\;C_{ij}$ are functions of the structural and vehicle properties, and their formulation is omitted here for brevity but can be found in [44].

Finally, by substituting the contact point displacement into Equation (2a), the displacement of the vehicle can be solved. Differentiating this displacement twice reveals the acceleration response of the vehicle:

(10) $$\begin{array}{c} {\ddot{q}}_{v}\left(t\right)=-\sum_{n=1}^{N}\sum_{i=1}^{2}D_{ij}\omega_{v}^{2}cos\left(\omega_{v}t\right)+E_{ij}{\left(\omega_{ji}-vk_{i}\right)}^{2}cos(\left(\omega_{ji}-k_{i}v\right)t)+\\ F_{ij}{\left(\omega_{ji}+vk_{i}\right)}^{2}cos\left(\left(\omega_{ji}+vk_{i}\right)t\right)+G_{ij}\left(2k_{i}\right)v^{2}cos\left(2k_{i}vt\right)+H_{ij} \end{array}$$

where

(11a) $$D_{ij}=\frac{2\left(v^{2}k_{i}^{2}\omega_{ji}^{2}+2\omega_{v}^{2}\right)v^{2}k_{i}^{2}M_{v}gb_{ij}}{\left(2k_{i}v-\omega_{v}\right)\left(2k_{i}v+\omega_{v}\right)\left(k_{i}v-\omega_{ji}-\omega_{v}\right)\left(k_{i}v+\omega_{ji}+\omega_{v}\right)\left(k_{i}v+\omega_{ji}-\omega_{v}\right)}$$

(11b) $$E_{ij}=\frac{-\omega_{v}^{2}k_{i}vM_{v}gb_{ij}}{2\omega_{ji}\left(k_{i}v-\omega_{ji}-\omega_{v}\right)\left(k_{i}v-\omega_{ji}+\omega_{v}\right)\left(k_{i}v+\omega_{ji}\right)\left(k_{i}v-\omega_{ji}\right)}$$

(11c) $$F_{ij}=\frac{\omega_{v}^{2}k_{i}vM_{v}gb_{ij}}{2\omega_{ji}\left(k_{i}v+\omega_{ji}+\omega_{v}\right)\left(k_{i}v+\omega_{ji}-\omega_{v}\right)\left(k_{i}v+\omega_{ji}\right)\left(k_{i}v-\omega_{ji}\right)}$$

(11d) $$G_{ij}=\frac{-\omega_{v}^{2}M_{v}gb_{ij}}{32\left(2k_{i}v+\omega_{v}\right)\left(2k_{i}v-\omega_{v}\right)\left(k_{i}v+\omega_{ji}\right)\left(k_{i}v+\omega_{ji}\right)}$$

(11e) $$H_{ij}=\frac{-M_{v}gb_{ij}}{2\left(k_{i}v+\omega_{v}\right)\left(k_{i}v-\omega_{v}\right)}$$

It is clear from Equations (10) and (11) that the frequencies of the rail-track-bridge system, $\omega_{ji}$, are included in the acceleration response of the vehicle, and therefore, theoretically speaking, can be extracted from the accelerations recorded on a sensor mounted on the moving mass. It can further be observed that the frequencies of the rail-track-bridge system are not observed in their original form in the acceleration response of the vehicle but they are shifted in the form of $\omega_{ji}\mp vk_{i}$ where $k_{i}=i\pi /L$.

### 2.2. Continuous Wavelet Transform

Traditionally, Fourier transform is widely applied to a signal to determine its frequency characteristics. However, it has two main drawbacks. First, it considers the entire signal and does not provide any information about the potential time-dependent variations in the frequency content of the signal. Furthermore, the Fourier transform is limited to sinusoidal waves as the basis for the decomposition of the signal. Wavelet transform (WT) alleviates both shortcomings of the Fourier transform by providing a detailed information about the variation of the frequency content with time as well as allowing different basis functions, called mother wavelets, to be used to decompose the signal. A mother wavelet is a waveform of limited duration and has an average value of zero:

(12) $$\int_{-\infty }^{+\infty }\Psi \left(t\right)dt=0$$

where Ψ(t) is the mother wavelet.

The Continuous Wavelet Transform (CWT) of a signal f(t) is given by:

(13) $$W\left(a,b\right)=\frac{1}{\sqrt{a}}\int_{-\infty }^{+\infty }f\left(t\right)\Psi \left(\frac{t-b}{a}\right)dt=0$$

W(a,b) represents how closely the signal at a given time interval is correlated with the wavelet. The scaling parameter, *a* is used to stretch and dilate the mother wavelet and is correlated to the frequency of the wavelet while the translation parameter, *b*, is used to move the wavelet of finite duration in time. In this study, Morlet wavelet [50] is used as it is widely used in structural health monitoring applications [48,51]. The reader is referred to these references for further information about wavelets [52].

## 3. Proposed Methodology

The proposed methodology consists of five steps:

1. Mount accelerometers on the bogeys of a train and measure the vibrations that occur before, during, and after the train crosses a bridge.
2. Compute the Fourier Amplitude Spectrum (FAS) of the recorded vibrations and identify the candidate frequencies. The candidate frequencies are the peaks that are visible in the FAS that can be the bridge frequencies.
3. Perform a continuous wavelet transform (CWT) of the acceleration signals recorded on the bogeys and create their wavelet coefficient maps.
4. For each candidate frequency, isolate the coefficients of the CWT for that frequency by taking a horizontal section of the wavelet coefficient map at the scale corresponding to that candidate frequency.
5. Compute the time variation of the energy of the coefficients at each candidate frequency and its development over time. The bridge frequency can then be identified as the frequency where the energy of the wavelet coefficients is concentrated at the time-frame where the bogey is crossing the bridge.

**Figure 2:**
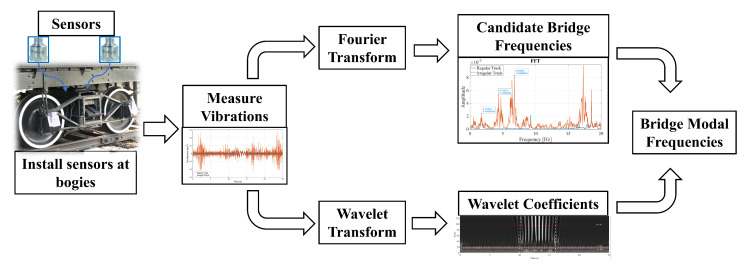
Flowchart of the proposed methodology.

The proposed methodology presented in Figure 2 is validated via case studies using a detailed finite element model of the train-track-bridge system.

## 4. Finite Element Model

### 4.1. Train Model

**Figure 3:**
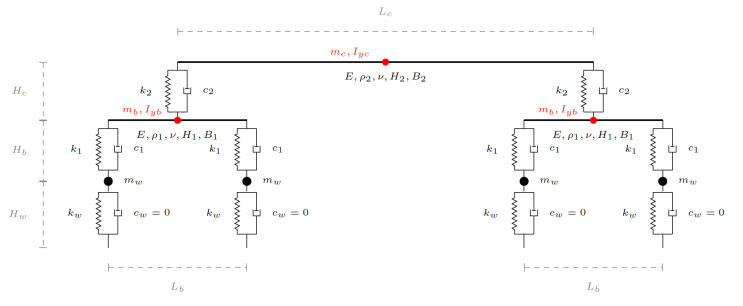
Schematic illustration of the train model and the parameters.

The train model properties used in the study are based on the Manchester train benchmark model [53]. A detailed three-dimensional model of the Manchester train was developed. The wheels were modeled using a linearly elastic spring and a lumped mass ($k_{w}$ and $m_{w}$ in Figure 3, respectively) while the bogies are modeled using a rigid beam supported by at the primary suspension system ($k_{1}$ and $c_{1}$) connected to the wheel and the bogie. Finally, the secondary suspension system ($k_{2}$ and $c_{2}$) connected to the bogie supports the car, which is modeled as a rigid beam. The principle of the model is explained in the schematic drawing shown in Figure 3 and the parameters used in the model and their values are listed in Table 1.

**Figure 4:**
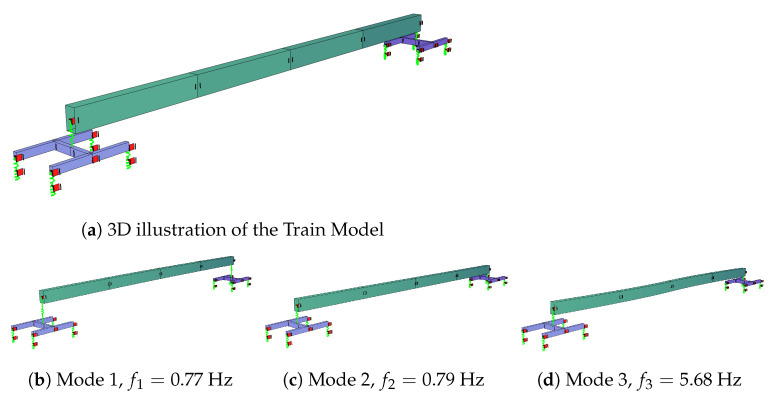
Numerical model of the train and its mode shapes.

The 3D train model, which is used in the numerical analysis, consists of two bogies (front and back) each housing four wheels; two on each side of the track. The car body is connected to the bogies at the middle of the bogie as illustrated in Figure 4a. Due to the geometry of the train model, the effect of the pitching motion on the vibrations recorded on the train-mounted sensors can be fully captured. On the other hand, the rolling motion of the train, although can be captured to a certain extent due to the 3D nature of the model, cannot be fully considered because the car body is modeled using a beam element.

Eigen-value analysis was conducted to determine the vibration frequencies and mode shapes of the train and the results are plotted in Figure 4b–d. The mode shapes and the vibration frequencies presented in Figure 4 indicate that the developed train model has vibration characteristics that are similar to their counterparts reported in the literature for the Manchester Train [53].

### 4.2. Bridge and Track Model

**Figure 5:**
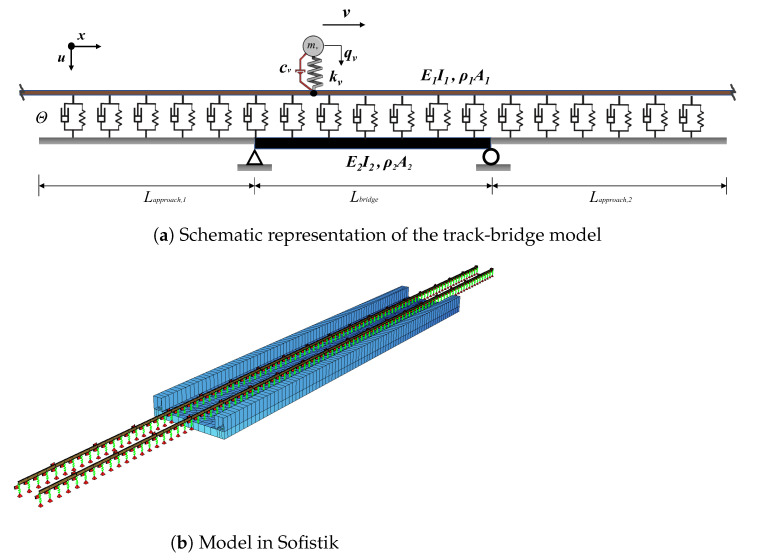
Numerical model of the track-bridge system.

The model developed in the Sofistik computational environment and used in the numerical analysis consists of three sections: the front approach, the bridge and the back approach. The front and the back approaches are modeled using elastic beams representing the rail supported by a set of springs and dashpots discretized at constant intervals, which represents the stiffness and damping characteristics of the track and the ballast. This rail and track model is continuous throughout the three sections of the model, i.e., the front and back approaches as well as the railway bridge. The lengths of the front and back approaches were selected as 58.8 m and 70.8 m, respectively. Figure 5 presents a 2D schematic view of the model as well as a 3D representation from the finite element software.

A typical prestressed concrete, single-span bridge with a span length of 32.4 m, was used in the study. The bridge deck has a U-shaped cross-section with a total depth of 1.4 m. It is 5.8 m wide and houses a single, ballasted track. The flanges of the cross section are 1.4 m high and 0.7 m wide, while the web is 0.5 m thick and 4.4 m wide. The area and the moment of inertia of its cross-section are 6.81 m${}^{2}$ and 16.89 m${}^{4}$, respectively. The longitudinal axis of the bridge is straight with no curvature. The Young´s Modulus and density of concrete was assumed to be 36 GPa and 25 kN/m${}^{3}$, respectively. The bridge deck was modeled using four-node shell elements.

**Figure 6:**
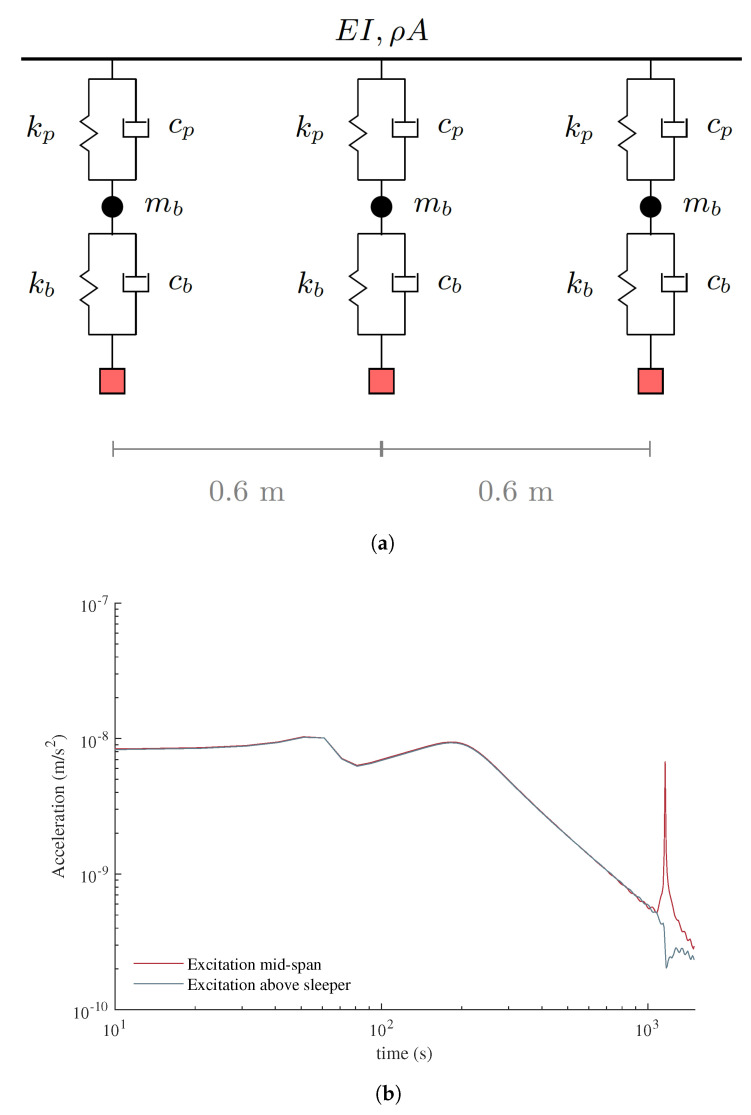
(**a**) Schematic illustration of the track model and (**b**) Receptance test results.

Figure 6a provides a detailed schematic illustration of the track model between three support points, i.e., three sleepers, on the track. The centerline distance between two sleepers was taken as 0.6 m, which is also equal to the unsupported length of the rail. The elastic Bernoulli–Euler beam representing the rail has the cross-section properties of the rail profile 60E1 and is supported by a pair of springs and dashpots at each support point. The spring-dashpot set at the bottom represents the stiffness and the damping provided by the ballast ($k_{b}$, $c_{b}$) while those at the top represents the stiffness and the damping of the rail-pad ($k_{p}$, $c_{p}$). In computing the stiffness and damping properties of the rail-pad, the rail-pad thickness was taken as 10 mm [54]. The mass of the ballast, $m_{b}$, is modeled as a lumped mass between the two elastic springs. The numerical values of the modeling parameters of the track are summarized in Table 2. The springs simulating the ballast are fully restrained at the bottom on the back and front approaches while, on the bridge, they are connected to elastic four-node shell elements that represent the bridge deck (Figure 5).

To validate the developed track model, a numerical receptance test was carried out on a 6 m long stretch. The receptance test is a widely used method to characterize the global track behavior for a range of frequencies and allows the identification of the main resonance of the rail system while quantifying its sensitivity to vibrations [55] as well as the dynamic flexibility of the track [56]. A sinusoidal load of 1N was applied at the middle of the 6 m rail with frequencies ranging from 10 Hz to 1500 Hz with 10 Hz increments. The sampling frequency was set to 6000 Hz. Acceleration time histories for each frequency was extracted from the model and the peak steady-state amplitudes were computed and plotted in Figure 6. Furthermore, plotted in Figure 6 is the results of the receptance test repeated by applying the sinusoidal load at the sleeper location. There are three main frequencies that can be observed from the receptance test at around f=60 Hz, f=200 Hz, and f=1160 Hz. The first frequency corresponds to the rails and sleepers oscillating on ballast and is thus associated with the ballast stiffness. The second frequency corresponds to the oscillation of the rails on the sleepers and depends on the stiffness of the rail-pads. On the other hand, the last frequency corresponds to the local bending of the rail elements between the sleepers and, thus, can only be captured when the load is applied at the mid-point of two sleepers. The receptance test results show that both the mode shapes and frequency of the rail-track system is in line with previous studies [57], thus validating the developed model.

**Figure 7:**
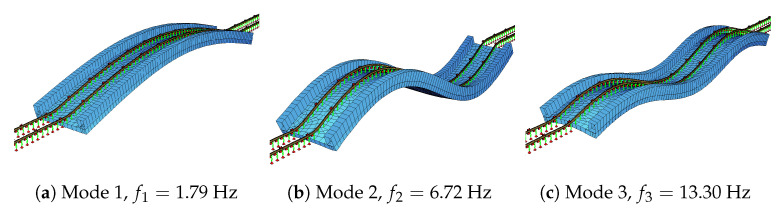
Mode shapes of the bridge.

Eigen-value analysis on the track-bridge model was conducted to determine the vibration frequencies and the mode shapes of the bridge. Accordingly, the natural frequencies of the first three modes were computed as 1.79 Hz, 6.72 Hz, and 13.30 Hz, respectively. The mode shapes and vibration frequencies are presented in Figure 7.

### 4.3. Track Irregularities

Track irregularities have a significant impact on the accelerations recorded on the trains and they adversely affect the indirect monitoring efforts by contaminating the frequency content of the acceleration signal [58]. As such, any method that aims to detect bridge frequencies from vehicle-mounted sensors needs to be robust against these adverse effects.

**Figure 8:**
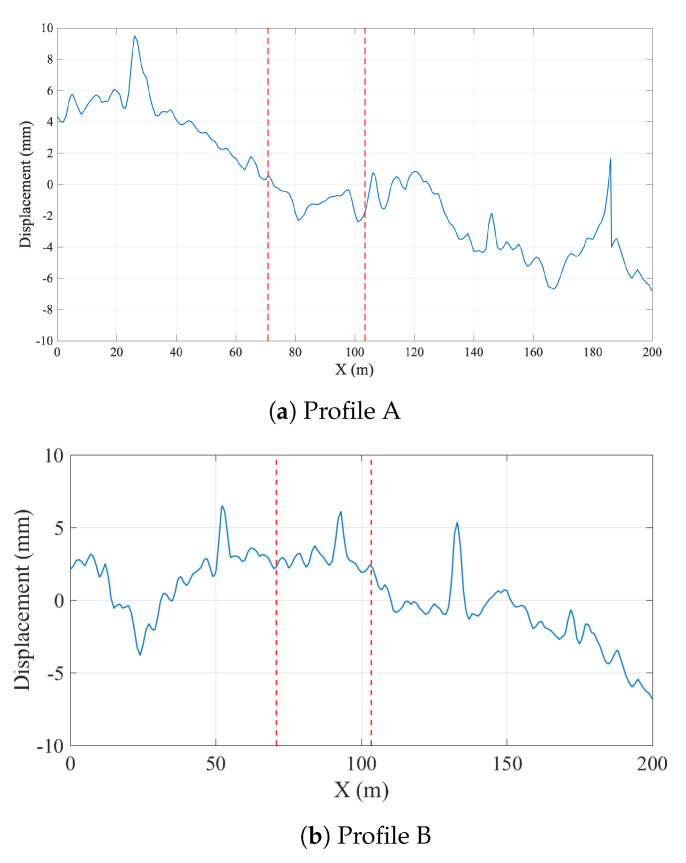
Track irregularity profiles.

In this study, two different track irregularity profiles, profiles A and B, which were recorded in Sweden, were used. The track profiles are plotted in Figure 8, where the two dashed red lines indicate the start and the end of the bridge, respectively. Irregularity profile A is a track profile is of good standard, whereas B is a track profile with average standard according to CEN/TC 256 [59]. As shown in Figure 8, the second profile represents a case where the irregularities are the most aggressive within the bridge while the first profile represents a case where the irregularities on the bridge are not as prominent.

## 5. Case Studies

Next, as summarized in the following subsections, the efficacy of the proposed methodology in identifying the bridge frequencies from vibrations recorded on train-mounted sensors is investigated on different case studies.

### 5.1. Case I: No Track Irregularities; Slow Train Speed

**Figure 9:**
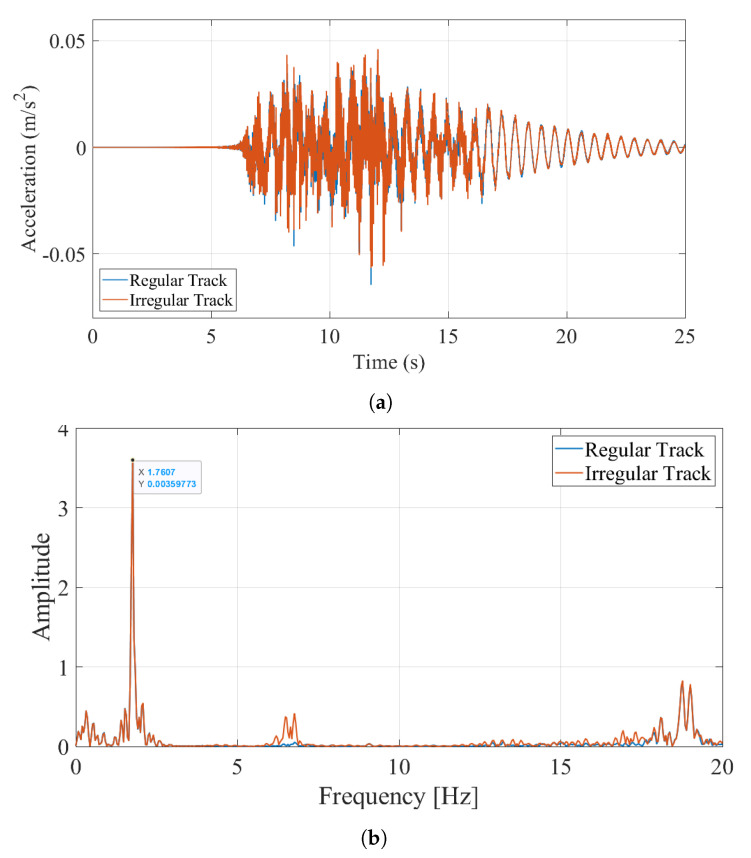
(**a**) Acceleration time history recorded at the x = 8.1 m on the bridge (**b**) Fourier Amplitude Spectrum of the recorded accelerations 20 km/h.

For the first case study, the Manchester train was driven on the track-bridge system with a speed of 20 km/h to be able to compute the vibrations on the bridge and on the train. The accelerations recorded at the quarter point of the bridge (i.e., 8.1 m from the left support) during the crossing of the train and its Fourier Amplitude Spectrum (FAS) is plotted in Figure 9. Both the time history and the FAS is plotted for two cases: ignoring and considering the track irregularities for the irregularity profile A. Figure 9a,b, respectively, indicate that neither the acceleration amplitudes nor the frequency content of the vibrations recorded on the bridge are significantly influenced by the track irregularities. As such, the first and second vibration frequencies of the bridge can be clearly identified from the Fourier amplitude spectrum for both cases.

**Figure 10:**
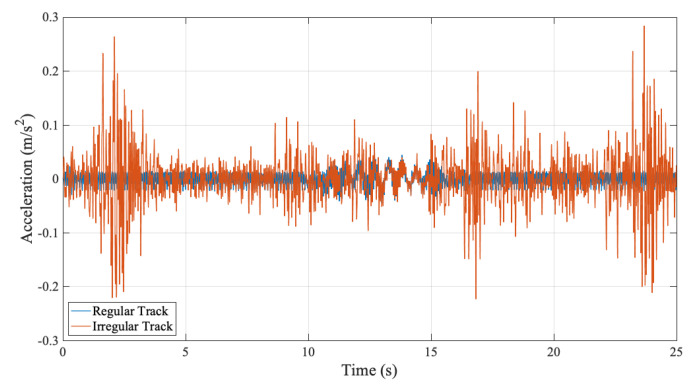
Accelerations recorded on the bogie for a speed of 20 km/h ignoring and considering track irregularities.

As the next step, the accelerations recorded at the middle of the back bogie ignoring and considering the track irregularities are plotted in Figure 10. The back bogie traverses the bridge between t = 10.48 s and t = 16.31 s. As expected, the track irregularities significantly impact the accelerations recorded on the bogie while the bogie is both on and off the bridge.

**Figure 11:**
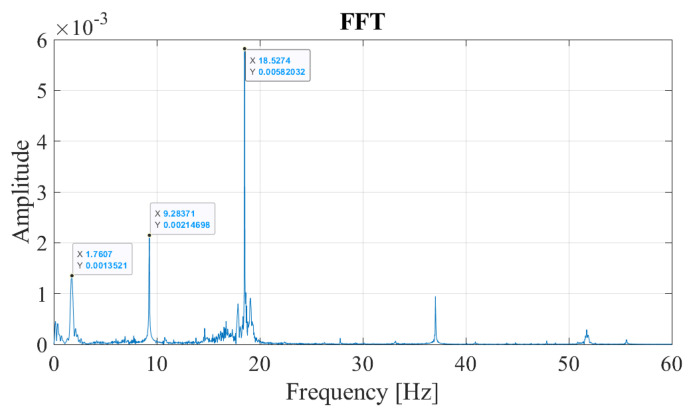
FAS of the accelerations recorded on the bogie ignoring track irregularities.

To evaluate the frequency content of the vibrations recorded on the bogie, the Fourier Amplitude Spectrum ignoring the track irregularities is plotted in Figure 11.

In addition to the first bridge frequency at f=1.76 Hz, there are several peaks on the FAS of the accelerations recorded on the bogie some having more energy than the first natural vibration frequency of the bridge. The first of these frequencies is visible at f=9.25 Hz. This frequency can be identified as the sleeper passing frequency for a speed of 20 km/h or 5.56 m/s, which is defined as the frequency that the bogie meets each consecutive sleeper that are spaced at every 0.6 m. Here, it should be noted that the shift in the first bridge frequency due to the driving frequency is not significant because of the relatively low speed of the vehicle.

**Figure 12:**
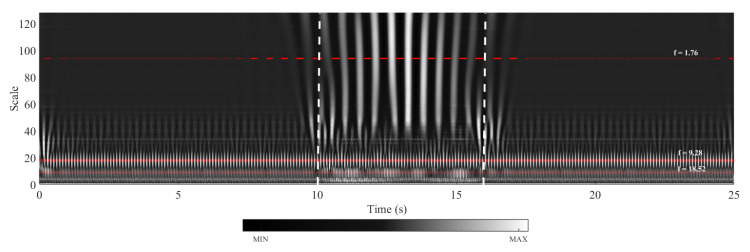
Wavelet coefficient map of the accelerations recorded on the bogie for a speed of 20 km/h.

Although the frequencies associated with the bridge and the train/track can be distinguished from the FAS plotted in Figure 11 when the train-track geometry and the speed of the train is known *a priori* as in the case of a numerical model, the FAS itself does not provide enough information to distinguish the bridge frequencies from the others when the track geometry and the train speed is unknown. The main reason for this is the fact that the Fourier Amplitude Spectrum is valid for the entire time history. On the other hand, the vibrations associated with the frequency of the bridge are expected to occur only while the sensor is traversing the bridge whereas the vibrations associated with the train-track system should be visible during the entire motion of the train including the front and back approaches. However, Fourier amplitude spectrum does not any provide any information on the time evolution of the frequency content of the vibrations. To overcome this shortcoming, Continuous Wavelet Transform of the acceleration signal recorded at the bogie using Morlet wavelet is computed and the wavelet coefficient map of the signal is plotted in Figure 12. The wavelet coefficient map shows that the high frequency vibrations indicated by scales lower than 20 in Figure 12 has a relatively evenly distributed energy content throughout the entire time history. On the other hand, the vibrations associated with lower frequencies (i.e., higher scales in Figure 12) are only prominent while the sensor is crossing the bridge. From this, it can be deducted that the lower frequencies prominent in the FAS (Figure 11) correspond to the bridge frequencies and the higher frequencies visible in the FAS plotted in Figure 11 correspond to the frequencies related to the vibrations in the train track system.

**Figure 13:**
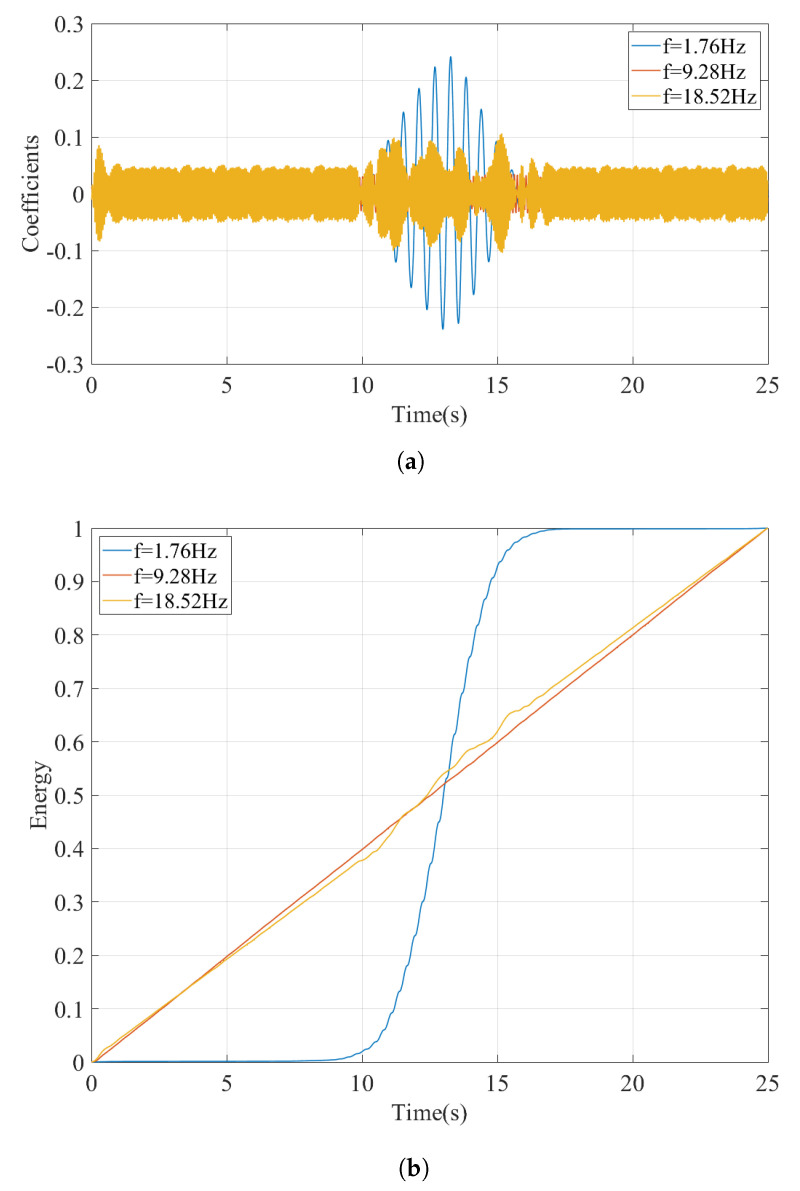
Regular track at a speed of 20 km/h. (**a**) Coefficients from CWT of the acceleration signal at different pseudo-frequencies. (**b**) Development of the energy in the coefficients at different pseudo-frequencies.

Although this visual identification can arguably be sufficient when the bridge frequencies and the frequencies related to the train-track system are well-separated, a more robust, objective, and theoretically sound method is necessary. For this, in this article, we propose to use the horizontal sections taken from the wavelet coefficient map. Physically, the horizontal sections of the wavelet coefficient map depict the time variation of the oscillations at each frequency by quantifying how close the signal is to the Morlet wavelet for that scale. The horizontal sections will be taken at the scales corresponding to the prominent frequencies observed in the Fourier amplitude spectrum of the signal selected using peak picking. Once they are selected, the scales corresponding to these frequencies can be computed by:

(14) $$a=\frac{f_{c}}{f_{s}\Delta }$$

where *a* is the scale corresponding to the frequency of interest, $f_{s}$, in Hz, $f_{c}$ is the center frequency of the wavelet in Hz, Δ is the time step of the data. In the example studied here, the Morlet wavelet with a center frequency of $f_{c}=0.825$ Hz is used and the sampling rate is Δ=0.005 s. The horizontal sections of the wavelet coefficient map taken at the scales corresponding to the frequencies of 1.76 Hz, 9.28 Hz, and 18.52 Hz, which are the prominent frequencies of the acceleration signal recorded at the bogie, are plotted in Figure 13a. It is clear from Figure 13a that the vibrations at the frequencies of 9.28 Hz and 18.52 Hz are continuous throughout the entire duration of the train’s journey on the front and back approaches as well as the bridge indicating that these frequencies are associated with the vibrations on the train-track system and not the bridge. On the other hand, the vibrations at the frequency of f=1.76 Hz occur only while the sensor is crossing the bridge and, therefore, this frequency can correctly be identified as the frequency of the bridge. This can be further visualized by computing the energy of the signals shown in Figure 13a and its evolution over time. For each time step, $t_{i}$, the ratio of energy in the coefficients signal $c_{f}\left(t\right)$ up to that time step to the total energy signal with a total duration of *T* is computed using:

(15) $$RE=\frac{\int_{0}^{t_{i}}{\left[c_{f}\left(t\right)\right]}^{2}dt}{\int_{0}^{T}{\left[c_{f}\left(t\right)\right]}^{2}dt}$$

where $t_{i}$ is each time step during the train traversing the entire track model including both approaches and the bridge; $0⩽t_{i}⩽T$, *T* is the total duration of the signal and $c_{f}\left(t\right)$ is the signal of the wavelet coefficients at a given frequency (scale). Computing the RE term for each of the coefficient signals in Figure 13a and plotting them as shown in Figure 13b, we can visualize the evolution of the energy in each signal during the crossing of the train. Recalling that the sensor crosses the bridge between $10.1\;s⩽t_{i}⩽16.0\;s$, it is clear that the entire energy in the signal for the frequency of 1.76 Hz is concentrated during the sensor crossing the bridge indicating that this frequency is associated with the vibrations on the bridge. On the other hand, the energy in the signals for 9.28 Hz, and 18.52 Hz evolve linearly during sensor’s entire journey over the approaches as well as the bridge indicating that the vibrations at these frequencies are related to the train-track system. Therefore, the steepness of the relative energy curve during the crossing of the bridge compared to that while the sensor is on the back and front approaches can be used to classify the peaks in the Fourier amplitude spectrum as bridge frequencies and frequencies associated with the train-track system.

### 5.2. Case II: Considering Track Irregularities; Slow Train Speed

To evaluate the efficacy of the proposed methodology when the track irregularities are considered, the numerical analysis was repeated by incorporating the track irregularity profiles shown in Figure 8. First, the acceleration response recorded on the bogie for track profile A (Figure 8a) will be investigated.

**Figure 14:**
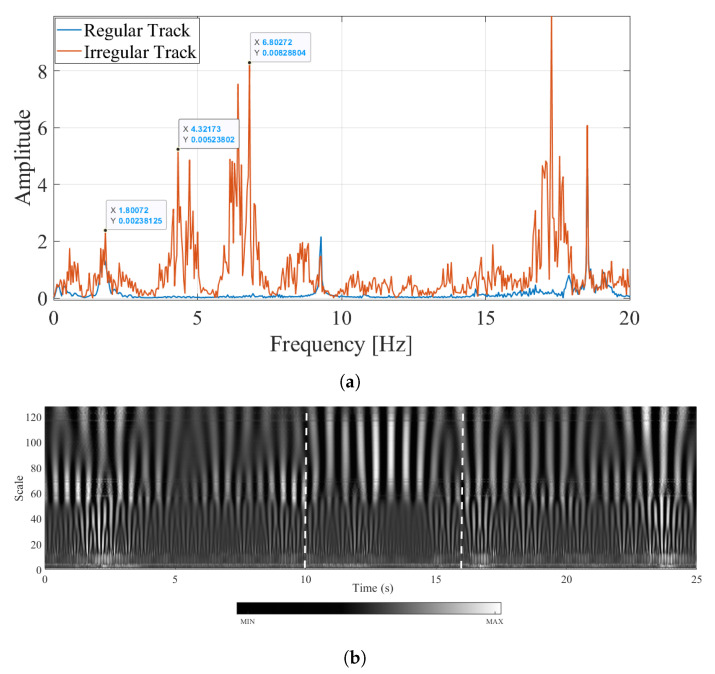
Frequency content of the accelerations recorded on the bogie for a speed of 20 km/h for the track irregularity profile A (**a**) Fourier Amplitude Spectrum (**b**) Wavelet Coefficient Map.

As the acceleration time history recorded on the bogie presented in Figure 10 indicates, track irregularities are the main source of the accelerations recorded on the bogie. The Fourier amplitude spectrum plotted in Figure 14a for regular and irregular tracks clearly demonstrates how complicated the frequency content becomes once the track irregularities are considered. However, a close inspection of Figure 14a clearly indicates that among the several peaks in the FAS, the bridge frequency is visible in the Fourier amplitude spectrum at f=1.80 Hz. This observation is in line with [44], which also showed that both analytically and numerically, the bridge frequency is visible in the FAS of the acceleration signals recorded on the bogie irrespective of the presence of the track irregularities.

To differentiate the bridge frequency from the other peaks in the FAS, the wavelet coefficient map of the acceleration signal recorded at the bogie for the irregularity profile A is created using continuous wavelet transform and plotted in Figure 14b. At the lower scales, i.e., the higher frequency region, the energy is concentrated at two intervals; 1.8 s $⩽t_{i}⩽$ 2.9 s, and 23.8 s $⩽t_{i}⩽$ 24.5 s. A quick inspection of the track irregularity profile (Figure 14b) show that these energy concentrations can be attributed to the two sudden bumps in the track for the profile A. On the other hand, the energy content in the lower frequency regions is more concentrated on the 10 s $⩽t_{i}⩽$ 16 s, i.e., while the sensor is crossing the bridge.

**Figure 15:**
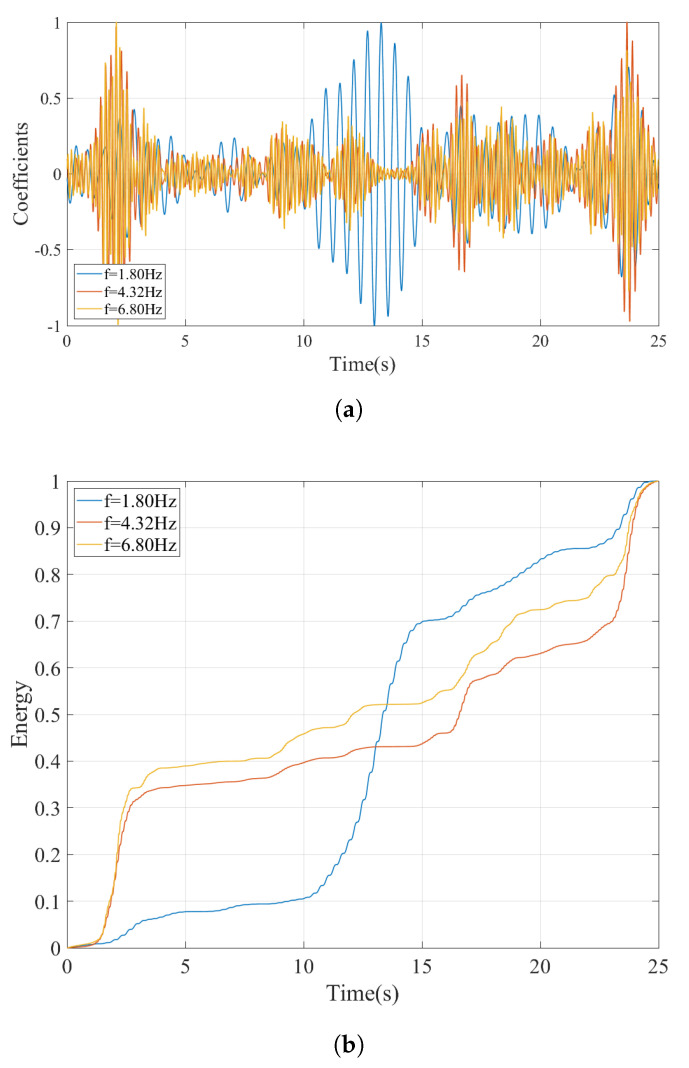
Irregular track (profile A) at a speed of 20 km/h. (**a**) Coefficients from CWT of the acceleration signal at different pseudo-frequencies. (**b**) Development of the energy in the coefficients at different pseudo-frequencies.

The proposed methodology requires that a horizontal section at the scales corresponding to each of the frequency peaks in the FAS. Considering the number of peaks in the FAS for the irregular profile shown in Figure 14a, only three such sections are presented at the frequencies of 1.80 Hz, 4.32 Hz, and 6.83 Hz in this article for brevity. These frequencies correspond to the highest peaks in the FAS of the acceleration response recorded at the bogie considering the track irregularity profile A. The time variation of the coefficients at these pseudo-frequencies are depicted in Figure 15a. While the coefficients at the frequency of f=1.80 Hz are dominated by the vibrations occurring as the sensor is crossing the bridge, the vibrations at the frequencies of 4.32 Hz, and 6.83 Hz are minimal during this phase (10 s $⩽t_{i}⩽$ 16 s). Instead, the vibrations associated with f=4.32 Hz and f=6.80 Hz are more dominant while the sensor is on the front and back approaches. The energy content of the coefficient signals computed using Equation (15) plotted in Figure 15b more clearly demonstrate where the energy at these frequencies are concentrated. The signals at all three pseudo-frequencies show a significant increase in the energy content at around t=2 s and again at around t=24 s, i.e., while the sensor is crossing the highest bumps on the rail profile A. However, the increase in the energy for f=1.80 Hz during the crossing of the bridge ($10s⩽t_{i}⩽16s$) is much higher compared to any other location indicating that the vibrations at this frequency are associated with the bridge response while the other two frequencies are associated with the train-track system. Although not presented here, repeating the procedure described above for all the peaks in the FAS diagram (Figure 14a) show that only the vibrations at f=1.80 Hz have the highest energy at during the crossing of the bridge. Hence, the frequency of f=1.80 Hz can be identified successfully as the bridge frequency using the proposed methodology among the other peaks of the FAS complicated significantly by the presence of track irregularities.

**Figure 16:**
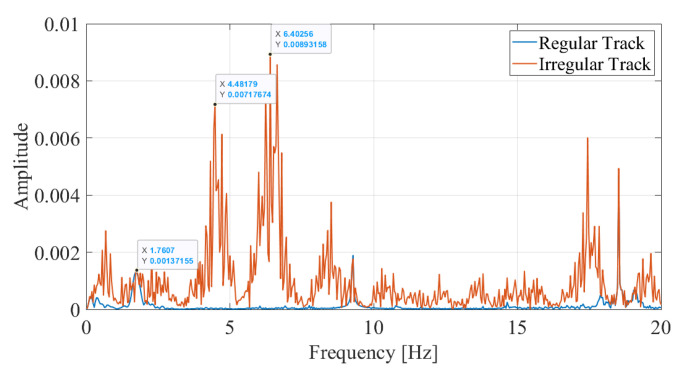
Fourier Amplitude Spectrum of vibrations recorded on the bogie for track profile B for V=20 km/h.

**Figure 17:**
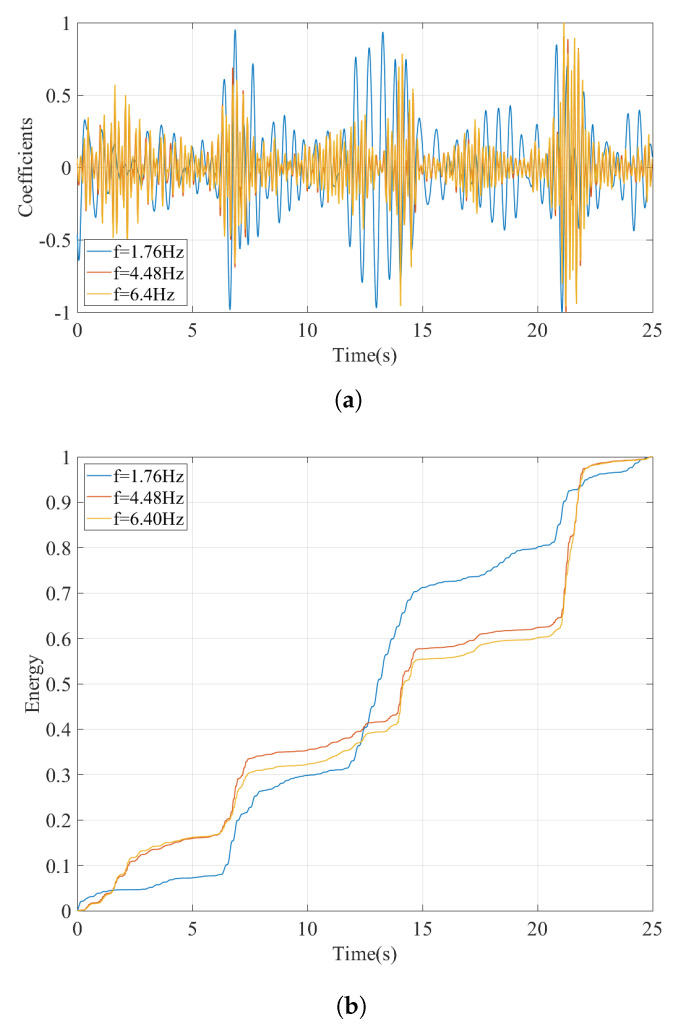
(**a**) Coefficient time history and (**b**) time evolution of energy at different frequencies computed using CWT for V=20 km/h for track irregularity profile B.

As the next step, the vibrations recorded on the bogie while the train is traversing the track profile B is evaluated. The Fourier amplitude spectrum depicted in Figure 16 shows that, as in the case of track irregularity A, several peaks can be observed, most of which can be attributed to the train-track vibrations and track irregularities. To be able to identify the bridge frequencies among these peaks, the wavelet coefficient map is created similar to the previous examples. Selecting three of the peaks at f=1.76 Hz, f=4.48 Hz, and f=6.40 Hz from Figure 16 as candidate frequencies, the time variation of the the wavelet coefficients at these frequencies are plotted in Figure 17a. Finally, the energy in the coefficient signals is computed and plotted in Figure 17b.

Comparing the evolution of the relative energy of the coefficient signals with time for in Figure 15b and Figure 17b for track profiles A and B, respectively, reveal that having an aggressive track irregularity on the bridge can further complicate the identification of the bridge frequency. More specifically, for all three frequencies selected from the Fourier amplitude spectrum, there are three points in time where a sharp increase in the relative energy is observed: 5.5 s $⩽t_{i}⩽$ 6.6 s, 14.2 s $⩽t_{i}⩽$ 14.8 s, and 21.0 s $⩽t_{i}⩽$ 22.5 s. These can be attributed to the three sudden bumps in the track irregularity profile, one of which is located on the bridge; see Figure 8b. However, a closer look at the relative energy curve in Figure 17b indicates that, for the frequencies of f=4.48 Hz and f=6.40 Hz, the highest increase in the relative energy is at 21.0 s $⩽t_{i}⩽22.5$ s, in other words while the sensor has already left the bridge. On the other hand, for the frequency of f=1.76 Hz, the increase in the energy while the sensor is on the bridge starts approximately 1.5 s earlier compared to its counterparts at f=4.48 Hz and f=6.40 Hz. Furthermore, this increase corresponds to almost 40% of the total energy in the signal, which is much higher compared to the other two points where the sharp increase in the relative energy while the sensor is on the bridge is limited to 15%. Finally, the time variation of the coefficients presented in Figure 17a show that the vibrations at f=1.80 Hz have a continuous and harmonic nature while the sensor is on the bridge (10.0 s $⩽t_{i}⩽$ 16.0 s) whereas the vibrations at f=4.48 Hz and f=6.40 Hz remain relatively low before the sensor reaches the end of the bridge. Therefore, it can be concluded that the frequency f=1.76 Hz is associated with the bridge vibrations while the frequencies f=4.48 Hz and f=6.40 Hz, although exhibit significant energy while the sensor is on the bridge, cannot be associated with the bridge vibrations because they are only limited to a certain point in the bridge while those at f=1.76 Hz are continuous. As such, the proposed methodology can successfully identify the bridge frequencies even for a track profile that has a sudden bump on the bridge.

### 5.3. Case III: Considering Track Irregularities; Higher Train Speeds

The studies that focus on system identification from vehicle-mounted sensors show that [58] increased train speeds significantly complicate the problem as the amount of recorded data is reduced. Most of the methodologies proposed so far are effective for very low speeds and fail to identify the bridge frequencies once the train speeds increase [11]. To be able to evaluate the efficacy of the proposed methodology for higher speeds, the numerical analysis was repeated for the speed of 90 km/h.

**Figure 18:**
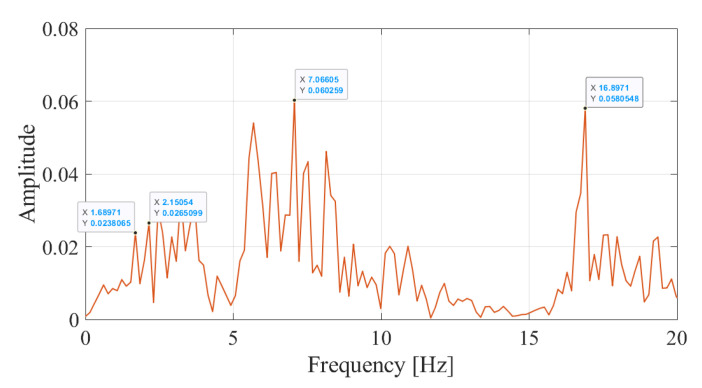
Fourier Amplitude Spectrum of the accelerations recorded on the bogie for a speed of 90 km/h.

Figure 18 depicts the Fourier amplitude spectrum of the accelerations recorded at the bogie for the speed of 90 km/h for the track irregularity profile A. Comparing the FAS for the case of regular track for speeds of 90 km/h and V=20 km/h (Figure 14a) demonstrates the additional complexity the higher train speeds bring into the frequency content of the vibrations. This is mainly due to the fact that, for the same sampling frequency and length of the railway section considered, the higher speeds lead to much less data compared to lower speeds, leading to inaccuracies in the frequency content of the data that can be recognized through Fourier transform.

**Figure 19:**
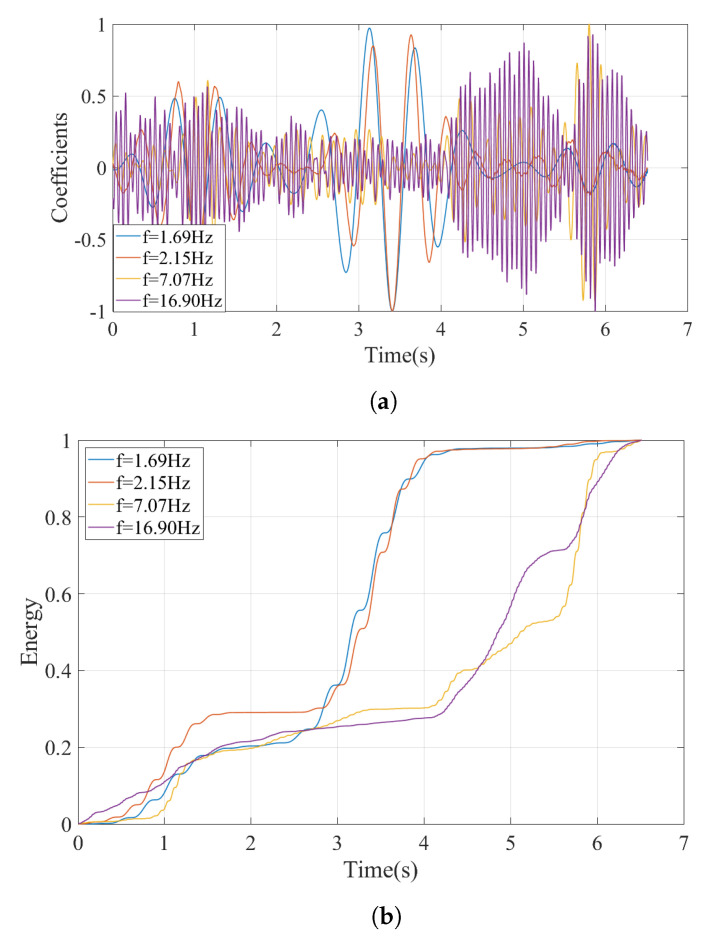
(**a**) Coefficients time history and (**b**) energy at different frequencies computed using CWT for V=90 km/h.

Despite this shortcoming, the FAS computed from the accelerations recorded considering the track irregularities and plotted in Figure 18 for a speed of V=90 km/h have two distinct peaks at f=1.69 and at f=2.15 Hz. Following the proposed methodology, the Wavelet Coefficient Maps of the acceleration records from the irregular track is created, and horizontal sections of the WCM at candidate frequencies are taken. For the speed of 90 km/h, four candidate frequencies were determined from Figure 18 as f=1.69 Hz, f=2.15 Hz, 7.07 Hz, and 16.90 Hz. The variation of the coefficients at these frequencies is plotted in Figure 19a and the evolution of the relative energy of the coefficient signals with time is plotted in Figure 19b. The back bogie which houses the sensor traverses the bridge between 2.3 s $⩽t_{i}⩽$ 4.1 s. Both the sharp increase in the energy of the coefficients signal for the frequencies of f=1.69 Hz and f=2.15 Hz during this time period and the coefficient signal itself clearly indicate that the vibrations at f=1.69 Hz and f=2.15 Hz are associated with bridge vibrations while the vibrations at the two other frequencies are associated with the train-track system as the energy at these frequencies are concentrated when the sensor is off the bridge.

The reason for observing two frequencies at a speed of 90 km/h instead of a single peak as in the case of V=20 km/h is the fact that the bridge frequency is evident in the acceleration response recorded in the bogie in a shifted form of $\omega_{ji}\mp vs.i\pi /L$ where *v* is the speed of the vehicle and *L* is the length of the bridge [44]. For a speed of 90 km/h (25 m/s) and L=32.4 m, this shift can be computed as 2.42 rad/s or 0.38 Hz. Accordingly, from the left shifted frequency (i.e., $\omega_{ji}-vs.i\pi /L$) of f=1.69 Hz, the bridge frequency can be computed as 2.08 Hz. From the right shifted frequency (i.e., $\omega_{ji}+vs.i\pi /L$) of f=2.15 Hz that is visible in Figure 18, the bridge frequency can be computed as 1.76 Hz. Here it should be noted that [44] reports that, although both left and right shifted frequencies theoretically are present in the acceleration response of the vehicle, only the right shifted frequency ($\omega_{ji}+vs.i\pi /L$) can be consistently recognized from the Fourier Amplitude Spectrum. Thus, the error in the left shifted frequency can be stated to be expected. As such, ignoring the left shifted frequency and focusing only on the right shifted frequency observed from the FAS, the bridge frequency can be computed as 1.76 Hz; an error of 1.7% compared to the computed frequency of 1.79 Hz.

Therefore, the proposed methodology distinguishes itself from the works reported in the literature by identifying the bridge frequency with relatively high accuracy for speeds which are in the range of regular travel speeds of trains en-route.

## 6. Conclusions

This article summarizes a new methodology to identify the vibration frequencies of a bridge from the vibrations recorded on the bogie of a train. First the theoretical background which shows that the vibration frequencies are indeed present in the accelerations on the vehicle traversing a railway bridge is revisited. The numerical simulations are based on detailed finite element models of the rail, track, bridge and the train. The train was modeled as a 3D MDOF system that can take the effects of pitching and, to a certain extent, rolling into account. The proposed framework distinguishes the bridge frequencies that are visible on the Fourier amplitude spectrum of the vibrations recorded on the bogie from the other prominent frequencies using continuous wavelet transform. The following conclusions can be drawn from the presented work.

- The numerical analysis indicate that the frequency content of the vibrations recorded on the bogie of a train is generally very complex with numerous peaks visible. This complexity is further amplified when the track irregularities are considered.
- Including the vibrations recorded on the front and back approaches in the data set collected from the bogie provides an opportunity to distinguish the bridge frequencies from the other peaks in the Fourier amplitude spectrum because the vibrations associated with the train-rail-track system are generally continuous through the entire route which includes the front approach, the bridge and the back approach. On the other hand, the vibrations on the bogie that are associated with the vibrations of the bridge occur only while the sensor is on the bridge.
- The continuous wavelet transform provides a powerful tool to distinguish the frequencies associated with the bridge vibrations from the other peaks visible in the Fourier amplitude spectrum. By taking a horizontal section on the wavelet coefficient map of the vibrations recorded on the bogie at the prominent frequencies visible on the FAS and computing the development of the energy at each frequency over time, the vibration frequencies associated with the vibrations of the bridge can be identified.
- The proposed method was shown to be able to pick out the bridge frequencies among other frequencies that have much higher energy than the bridge frequencies.
- Two different track irregularity profiles used in the numerical analysis showed that the proposed method can successfully detect the bridge frequencies even for very aggressive track irregularity profiles with sudden bumps located in the middle of the bridge.
- The proposed method was shown to work as effectively for high speeds as well as low speeds. As shown theoretically, the bridge frequencies that can be detected on the vehicle is shifted and this shift is linearly proportional to the speed of the vehicle. As long as this shift is considered, the bridge frequencies can be identified successfully for a speed of 90 km/h. The capability of identifying the bridge frequencies for relatively high train speeds distinguishes the proposed method from most of the work in the literature that is limited only to low vehicle speeds.
- The identified bridge frequencies are limited to the first vibration mode. This can be explained by the fact that the behavior of single-span bridges is generally dominated by the first mode. As such, only the first mode frequency is visible in the FAS of the accelerations recorded on the bogie. To evaluate the efficacy of the proposed method in identifying higher mode frequencies, further analysis on bridges whose behavior is significantly influenced by higher modes is needed.

The study presented herein is limited to single-span bridges. Although this limitation is similar to the vast majority of similar literature, further work on multi-span bridges with vehicle models that represent en-route trains are required to be able to fully realize the potential of vehicle-mounted sensors operating on railway bridges. Furthermore, although a very detailed vehicle model is used in this study, it was limited to a single locomotive. Furthermore, the proposed method could only identify the first mode frequency of the bridge. Future studies should address this disadvantage by developing methods that can identify higher mode frequencies. Finally, only the Morlet wavelet was used in the current article. However, other wavelets such as Debuchies, Coiflet and Gaussian have previously been used successfully in structural health monitoring applications [60] and their efficacy in distinguishing the bridge frequencies when used within the proposed framework should be investigated.

## Figures and Tables

**Table 1:** Mechanical properties of the train components.

Component	Property	Symbol	Value
Wheel	Stiffness	$k_{w}$	$1\times 10^{5}$ kN/m
	Mass	$m_{w}$	906.5 kg
	Height	$H_{w}$	0.46 m
Boogie	Stiffness	$k_{1}$	1120 kN/m
	Damping	$c_{1}$	4 kNs/m
	Mass inertia	$I_{yb}$	1610 kgm^2^
	Mass	$m_{b}$	2615 kg
	Height	$H_{b}$	0.88 m
	Length	$L_{b}$	2.56 m
	Density	$\rho_{1}$	10,200 kg/m^3^
	Young’ Modulus	*E*	$1\times 10^{5}$ GPa
	Poison’s ratio	ν	0.2
	Cross section	$H_{1},B_{1}$	0.25 m, 0.15 m
Car	Stiffness	$k_{2}$	430 kN/m
	Damping	$c_{2}$	20 kNs/m
	Mass inertia	$I_{yc}$	$1.97\times 10^{6}$ kgm^2^
	Mass	$m_{c}$	32,000 kg
	Height	$H_{c}$	1.8 m
	Length	$L_{c}$	19 m
	Density	$\rho_{1}$	7400 kg/m^3^
	Cross section	$H_{2},B_{2}$	0.65 m, 0.35 m

**Table 2:** Mechanical properties for track components.

Component	Property	Symbol	Value
Rail-pad	Stiffness	$k_{p}$	62 MN/m
	Damping	$c_{p}$	32 kNs/m
ine Ballast	Stiffness	$k_{b}$	230 MN/m
	Damping	$c_{b}$	200 kNs/m
	Mass	$m_{b}$	1400 kg
Rail	Young’s modulus	$k_{a}$	200 GPa
	Poisson’s ratio	$\nu_{r}$	0.3
	Area	$A_{r}$	$8.13\times 10^{-3}$ m^2^
	Moment of Inertia	$I_{y}$	$3.09\times 10^{-5}$ m^4^
	Density	$\rho_{r}$	7800 kg/m^3^

## Data Availability

The data generated through numerical simulations is available upon request.
